# Congenital Hypothyroidism among Infants Undergoing Thyroid Function Test in a Tertiary Care Centre: A Descriptive Cross-sectional Study

**DOI:** 10.31729/jnma.7505

**Published:** 2022-06-30

**Authors:** Bijaya Mishra, Nisha Keshary Bhatta, Mohan Chandra Regmi, Binod Kumar Lal Das, Seraj Ahmed Khan, Basanta Gelal, Apeksha Niraula, Madhab Lamsal

**Affiliations:** 1 Department of Biochemistry, B.P. Koirala Institute of Health Sciences, Buddha Road, Dharan, Nepal; 2 Department of Paediatrics and Adolescents Medicine, B.P. Koirala Institute of Health Sciences, Buddha Road, Dharan, Nepal; 3 Department of Obstetrics and Gynaecology, B.P. Koirala Institute of Health Sciences, Buddha Road, Dharan, Nepal

**Keywords:** *congenital hypothyroidism*, *Nepal*, *newborn screening*, *prevalence*

## Abstract

**Introduction:**

Congenital hypothyroidism is the most preventable and treatable cause of mental retardation in newborns and infants. Screening for congenital hypothyroidism in newborns and infants is not a routine practice in our part of the world. This study aimed to find out the prevalence of congenital hypothyroidism among infants undergoing thyroid function test in a tertiary care centre.

**Methods:**

A descriptive cross-sectional study was done in the Department of Biochemistry, from laboratory records starting 14^th^ April, 2013 to 13^th^ April, 2020 after obtaining ethical clearance from the Institutional Review Committee (Reference number: 1502/019). A total of 1243 infants whose thyroid function tests were performed were obtained using convenience sampling. Thyroid-stimulating hormone was categorised per the European Society of Paediatric Endocrinology guidelines. Data were entered and analysed using Microsoft Excel 2011 and the Statistical Package for the Social Sciences version 11.5. Point estimate at a 95% Confidence Interval was calculated along with frequency and percentages for binary data.

**Results:**

Among 1243 infants, 56 (4.50%) (3.35-5.65 at 95% Confidence Interval) infants were diagnosed with congenital hypothyroidism.

**Conclusions:**

The prevalence of congenital hypothyroidism was higher than other studies done in similar settings. An unexpected finding of treatment-induced hyperthyroidism was observed, indicating a lack of regular and timely follow-up of infants diagnosed with congenital hypothyroidism.

## INTRODUCTION

Congenital Hypothyroidism (CH), if diagnosed and treated early, is one of the most preventable and treatable causes of mental retardation in newborns and infants. Due to the passage of maternal thyroid hormone through the placenta to some extent, clinical features of CH remain subtle rendering newborns undiagnosed at birth.^1^ Studies from various countries indicated treatment of CH should be started no later than the first 2 weeks of life.^2^

Newborn screening for CH is not a routine practice in Nepal, thus studies regarding thyroid function testing in neonates and infants are very limited from our context. B.P. Koirala Institute of Health Sciences (BPKIHS), is one of the tertiary care centres in Eastern Nepal. However, prevalence and baseline data regarding CH screening among infants are not available from our context.

This study aimed to find out the prevalence of congenital hypothyroidism among infants undergoing thyroid function test in a tertiary care centre.

## METHODS

A descriptive cross-sectional study was conducted in the Department of Biochemistry, at the BPKIHS, Nepal. Ethical clearance was obtained from the Institutional Review Committee (Reference number: 1502/019). The data for the study was collected from hospital-based data from Biochemistry Immunoassay Laboratory for Thyroid Function Test (TFT) records starting from 14^th^ April, 2013 to 13^th^ April, 2020. The available data on thyroid function testing on infants (day 1-day 365) were collected using the convenience sampling technique. As a hospital-based mode of data collection, all clinical history was not available in the records, however, those with confirmed preterm delivery were excluded. The sample size was calculated using the following formula:


$$\begin{array}{ll} \text{n} & ={\text{Z}}^{2}\times \frac{\text{p}\times \text{q}}{{\text{e}}^{\text{2}}}\\ & =1.96^{2}\times \frac{0.5\times 0.5}{0.03^{2}}\\ & =1067 \end{array}$$

Where,

n = minimum required sample size
Z = 1.96 at 95% Confidence Interval (CI)
p = prevalence taken as 50% for maximum sample size
q = 1-p
e = margin of error, 3%

Adding 10% missing record rate, the sample size was 1186. However, 1243 samples were taken.

Biochemical criteria for treatment initiation in infants with high Thyroid Stimulating Hormone (TSH) as per European Society of Paediatric Endocrinology (ESPE) guidelines have given the cut-off values on TSH in regards to Dried Blood Spot (DBS) sampling and venous blood sampling.^2^ As DBS testing facility is unavailable in Nepal, venous blood sampling was used for thyroid hormone assay in our study. The thyroid function test parameters were performed on SNIBE Maglumi 2000 Chemiluminescence Analyzer. All the data obtained were categorised as per ESPE guidelines on the basis of a TSH value in a venous sample.^2^

Data were entered and analysed using Microsoft Excel 2011 and the Statistical Package for the Social Sciences version 11.5. Point estimate at a 95% CI was calculated along with frequency and percentages for binary data.

## RESULTS

Among 1243 infants, 56 (4.50%) (3.35-5.65 at 95% Confidence Interval) infants were diagnosed with congenital hypothyroidism. Among 56 patients with congenital hypothyroidism, 32 (57.14%) were male infants and 24 (42.86%) were female. Thirty-six (64.28%) babies diagnosed with hypothyroidism were between 0-6 months of age (Table 1).

**Table 1:** Thyroid hormone status among different age groups (n = 56).

Age (months)	Congenital hypothyroidism n (%)
0-3	18 (32.14)
3-6	18 (32.14)
6-9	10 (17.86)
9-12	10 (17.86)

Forty-two (75.00%) infants under the hypothyroidism category were diagnosed with CH under treatment (Table 2). In infants with CH, there were 12 (21.43%) infants who had low TSH value (0.03 μIU/mL).

**Table 2:** Clinical history among the infants with a diagnosis of Congenital Hypothyroidism (n = 56).

History	Congenital hypothyroidism n (%)
Mother with hypothyroidism	2 (3.57)
Baby diagnosed as CH	42 (75.00)
Prolonged jaundice	2 (3.57)
Clinical history missing in record	9 (16.07)
Others	1 (1.78)

## DISCUSSION

Thyroid hormone with an important role in the development of a central nervous system, thyroid hormone deficiency at birth is called CH.^3^ As the clinical features of CH as subtle or absent at birth, many newborn babies remain undiagnosed.^4^ Pilot screening programs for CH were developed in many part of the world by using newborn heel prick filter paper dried blood samples, and have now been established in most parts of the world.^5^ Newborn Screening (NBS) for CH has been considered a major achievement in the field of preventive medicine and is widely accepted as a tool in primary health care for infants like breastfeeding, immunisation and oral rehydration.^6^

BPKIHS is one of the largest tertiary care centres in Eastern Nepal, with an average of 10,000 live births per year. Lack of an established health policy to screen newborns for CH, lack of convenient heel prick facility and testing, parents reluctant for phlebotomy in their babies and lack of awareness regarding the significance and benefit of CH screening in newborns could be the reason behind the paucity of CH screening in our context.

The prevalence of many metabolic disorders in Nepal is not yet known.^7^ As a common preventable cause of mental retardation and growth deficiency in a paediatric population, the overall incidence of CH ranges from 1 in 3000 to 1 in 4000 live births in different parts of the world.^8^ The incidence was reported as 1 in 2500 in a pilot study done in Nepal.^7^

There is an inverse relationship between intelligence quotient and the age of diagnosis, with studies indicating treatment of CH starts no later than the first two weeks of life.^2,9^ A study done in Nepal has shown that the majority of babies with CH were detected at the age of 6 to 36 months of life when parents noticed some form of delay in developmental milestones.^10^ In our study, the babies diagnosed in other age categories i.e. after 3 months, intellectual development might be affected to some extent due to delay in diagnosis and lack of regular follow-up for thyroid hormone monitoring.

The most sensitive test for detecting primary CH is a measurement of Thyrotropin (TSH).^11^ As per the ESPE Guidelines, utilising the TSH value, we categorised 1.3% of infants under CH, in addition, there were 3.2% of infants already diagnosed as CH (excluding data counted in ESPE category). Thus, the prevalence of CH in infants was 4.5%. We could not find literature on the prevalence of CH in our context for comparison. However, a study done in similar set-up had shown the prevalence of CH to be as 1.4%.^12^ The prevalence was reported high in our study probably because of testing done in high clinical suspicion in addition to diagnosed cases under treatment monitoring. Considering the prevalence, early diagnosis and treatment of CH through newborn screening can prevent neurodevelopmental delay and optimise its developmental outcome, hence CH screening in this population is highly warranted.^11^

Follow-up and ruling out of CH would assure both the clinician and parents to some extent that CH would not pose a threat of fatal neurodevelopmental consequences for the baby in later life. In a study done in India, out of 1530 neonates screened none had CH, however, 204 neonates had borderline TSH values out of which 163 neonates came for follow-up at day 21 of birth and were ruled out for CH, with a probable diagnosis of transient hypothyroidism.^13^ Similar retesting and ruling out, and follow-up data were missing in our study. Close collaboration between laboratory and treating specialists is necessary to ensure adequate treatment and follow-up of babies identified by CH screening programs.^14^

Of the total 56 infants with CH, 12 (21.43%) infants had low TSH value (<0.03 μ IU/mL). This indicates treatment-induced hyperthyroidism in these infants. Lack of timely follow-up and regular thyroid hormone monitoring might be the reason behind this finding. However, due to the lack of proper clinical history of all infants and mothers in the laboratory records we were not able to address the exact reason for all infants with low TSH values. Future studies are needed to address and confirm this issue in detail from our context. With a clear-cut benefit of early diagnosis and treatment of CH, and recommendation for its screening to be introduced worldwide, we found that TSH testing was not performed exclusively as a part of a newborn screening program in our context and infants were only tested in a mere clinical suspicion or clinical history indicating testing. This would lead to a substantial delay in diagnosis hence affecting neurocognitive development.

Having a retrospective approach to data collection, we were not able to gather all the relevant information about infants and mothers. As this was a descriptive crosssectional study, we could not find out the association between the variables.

## CONCLUSIONS

The prevalence of congenital hypothyroidism was higher than other studies done in similar settings. Newborn screening for CH is a routine practice in most parts of the world. TFT in infants was performed in presence of clinical suspicion or specific indication and was not performed as a part of exclusive newborn screening practice. We were able to generate baseline statistics on CH screening practice from our context. A larger prospective study on universal screening of newborns for CH is highly recommended.
